# Dataset on factors influencing pedestrian crosswalk usage behavior in high-density urban areas of a developing country

**DOI:** 10.1016/j.dib.2024.110912

**Published:** 2024-09-04

**Authors:** Nazmus Sakib, Tonmoy Paul, Md. Tawkir Ahmed, Khondhaker Al Momin, Saurav Barua

**Affiliations:** a Department of Civil Engineering, Ahsanullah University of Science and Technology, Dhaka, Bangladesh; b Department of Civil Engineering, Dhaka University of Engineering and Technology, Bangladesh; c Department of Civil Engineering, Daffodil International University, Dhaka, Bangladesh

**Keywords:** Pedestrian safety, Developing country, Safe crosswalk, High-density urban area, Questionnaire survey

## Abstract

The dataset consists of survey data on pedestrian crosswalk usage behavior in high-density urban areas of a developing country, specifically collected from Dhaka, the capital city of Bangladesh. Data were gathered through a questionnaire survey conducted at twelve key locations, covering eight attributes related to crosswalk behavior and the demographic details of respondents. The survey yielded 682 valid responses, focusing on factors such as the suitability of crosswalk locations, guard rails, and lighting. The dataset is structured to support analyses using supervised machine learning techniques, facilitating reproducibility, secondary analysis, and policy development for pedestrian safety improvements. Furthermore, the dataset can be reused for cross-validation of future studies, comparison with pedestrian behavior in similar urban settings, and the development of predictive models to enhance pedestrian infrastructure in other developing regions.

Specifications Table

**Table:** 

Subject	Planning and development
Specific subject area	A data-in-brief article presenting survey data on pedestrian crosswalk usage behavior and safety in high-density urban areas Dhaka, Bangladesh
Type of data	Table Raw Data (.xls), Analyzed and Descriptive.
Data collection	A face-to-face questionnaire survey was conducted at twelve significant locations (Baridhara, Banani, Gulshan-2, Badda, Bijoy Sarani, Panthapath, Saarc Fowara, Mouchak, Shantinagar, Dhanmondi, Shahabag, and Kakrail Circle) to understand pedestrian preference regarding crosswalk usage in high-density urban areas like Dhaka City. The study area's geographical coordinates range from 23°42′N to 23°54′N latitude and 90°20′E to 90°28′E longitude. Footpaths near intersections and roundabouts were visited on various weekdays to ensure representation of pedestrians from across Dhaka City. Initially, a pilot survey was conducted with 50 pedestrians from diverse demographic backgrounds to test and refine the questionnaire, ensuring its relevance and comprehensiveness. The feedback this pilot survey helped identify and resolve any ambiguities or issues in the questions. The main survey focused on eight attributes and demographic characteristics (Gender, Age, Crosswalk Usage Frequency, and Crosswalk Preference), using a 5-point Likert scale. A total of 700 responses were collected, and after data screening, the sample size was reduced to 682.
Data source location	Baridhara (Dhaka, Bangladesh) Banani (Dhaka, Bangladesh) Gulshan-2 (Dhaka, Bangladesh) Badda (Dhaka, Bangladesh) Bijoy Sarani (Dhaka, Bangladesh) Panthapath (Dhaka, Bangladesh) Saarc Fowara (Dhaka, Bangladesh) Mouchak (Dhaka, Bangladesh) Shantinagar (Dhaka, Bangladesh) Dhanmondi (Dhaka, Bangladesh) Shahabag (Dhaka, Bangladesh) Kakrail Circle (Dhaka, Bangladesh)
Data accessibility	Repository name: Mendeley Data identification number: 10.17632/rr7mfjftx2.2 Direct URL to data: https://data.mendeley.com/datasets/rr7mfjftx2/2
Related research article	Sakib, N., Paul, T., Ahmed, Md. T., Momin, K. A., & Barua, S. (2024). Investigating factors influencing pedestrian crosswalk usage behavior in Dhaka city using supervised machine learning techniques. Multimodal Transportation, 3(1), 100,108. DOI: https://doi.org/10.1016/j.multra.2023.100108

## 1 Value of the Data

• These data provide valuable insights into pedestrian crosswalk usage behavior in high-density urban areas of a developing country, helping to identify key factors influencing pedestrian decisions.
• The dataset serves as an essential resources for policymakers, institutional leaders, and transportation safety specialists to develop policies aimed at enhancing pedestrian safety. By understanding the perceptions and challenges faced by pedestrians, more effective policies can be formulated to encourage the use of crosswalks.
• The dataset enables comparisons between pedestrian behaviors in different high-density urban environments of developing countries, facilitating cross-cultural studies and enhancing the generalizability of findings.
• Researchers can reuse this dataset to develop and validate machine learning models aimed at predicting pedestrian behavior and improving pedestrian safety in similar urban settings. Overall, the dataset contributes to the body of knowledge on pedestrian safety and crosswalk usage behavior from the perspective of pedestrians.

## 2 Background

The primary goal of the survey is to address knowledge gaps and inform policymaking on pedestrian crosswalk usage behavior. Pedestrians were interviewed face-to-face, with questions focusing on their demographic profiles and perceptions of crosswalk usage. The survey was designed to explore factors affecting crosswalk usage behavior, as identified in our study, which investigates factors influencing pedestrian crosswalk usage behavior in Dhaka city using supervised machine learning techniques [1]. The goal is to develop a comprehensive framework for understanding crosswalk usage behavior from the pedestrian's perspective. The dataset provides valuable insights that can aid in the development of targeted interventions to enhance pedestrian safety and infrastructure in similar urban settings globally.

## 3 Data Description

The survey data aimed to investigate the factors influencing pedestrian's crosswalk usage behavior in Dhaka city. Data were collected through a face-to-face questionnaire survey conducted from May 2020 to July 2020 at twelve critical locations, including Baridhara, Banani, Gulshan-2, Badda, and others. The questionnaire was divided into two sections: Section A focuses on demographic characteristics (Gender, Age, Crosswalk Usage Frequency, and Preference for Crosswalk Usage), while Section B comprised close-ended questions about pedestrian crosswalk usage behavior.

A total of 700 participants completed the paper-based questionnaire. After screening for incomplete responses and outliers, the final sample size was reduced to 682, which exceeds the calculated minimum sample size of 384, determined using the following equation [1,2].

(1) $$n\;=\frac{Z^{2}}{4E^{2}}$$

Here, n represents the sample size for pedestrians. At a 95 % confidence level, the Z score is 1.96, and the maximum acceptable error (E) was set at 0.05, considering the time constraints of the pedestrians. Due to the use of convenience sampling methods, the dataset may not be fully representative of the entire pedestrian population in Dhaka city.

Respondents indicated their level of agreement with statements about crosswalk usage behavior on a 5-point Likert scale, ranging from Strongly Disagree (1) to Strongly Agree (5). The Raw data, which includes these responses, and demographic details, is available in the dataset (Survey data on pedestrian crosswalk usage behavior in Dhaka, Bangladesh.xlsx) along with the survey questionnaire (Pedestrian Crosswalk Usage Behavior Survey.docx) at https://data.mendeley.com/datasets/rr7mfjftx2/2

**Fig. 1:**
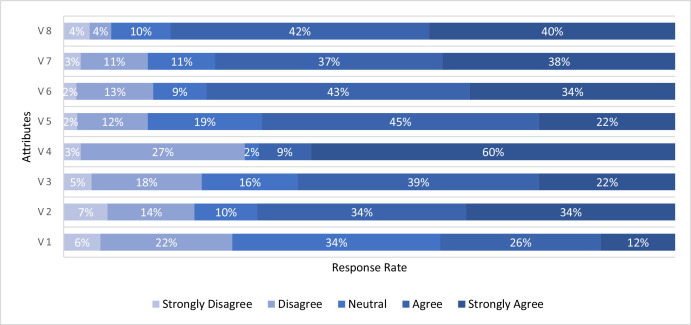
Survey Response of participants on crosswalk usage behavior. Source: [1].

**Fig. 2:**
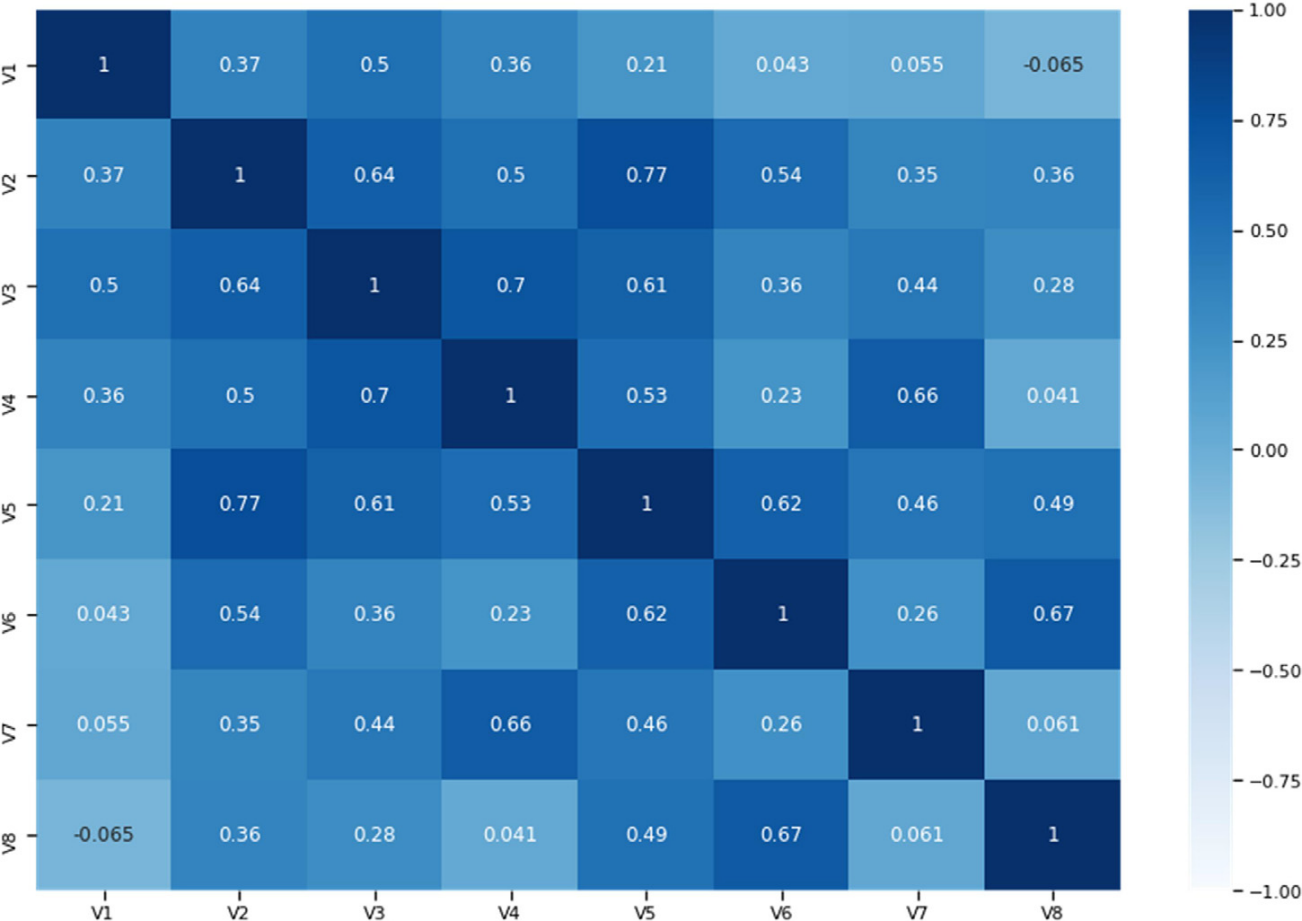
Spearman's Correlation Matrix. Source: [1].

Table 1 presents the demographic characteristics of the sample corresponding to Section A of the questionnaire. Table 2, corresponding to Section B, provides descriptive statistics of the crosswalk usage behavior attributes, their definitions, and the reliability of these attributes using Cronbachʼs alpha. Fig. 1 displays the survey responses related to crosswalk usage behavior. Spearman's correlation test was conducted to check for multicollinearity among the attributes, with an absolute correlation coefficient greater than 0.8 indicating multicollinearity [3]. As shown in Fig. 2, no correlation coefficients exceed 0.8, confirming that the eight attributes are distinct and independent. Additionally, Kaiser–Meyer–Olkin (KMO) and Bartlett's tests were conducted to assess the reliability of the survey data. The KMO value was 0.827 (≥ 0.8), and Bartlett's Test of Sphericity results were Chi-square = 3190.185, df = 28, *p* < 0.001 [4]. These results indicate that the questionnaire is well-constructed, and the collected data is appropriate for analysis.

**Table 1:** Descriptive statistics of participants’ demographic characteristics (*n* = 682).

Variables		Frequency	Percentage
**Gender**	Female	63	9.24
	Male	619	90.76
**Age (years)**	<20	5	0.7
	20–40	644	94.4
	40–60	16	2.3
	>60	17	2.5
**Crosswalk Usage Frequency**	Very rarely	71	10.4
	Once or twice a month	27	4.0
	Once or twice a week	341	50.0
	Everyday	156	22.9
	More than once a day	87	12.8
**Crosswalk Usage Preference**	Yes	640	93.8
	No	42	6.2

**Table 2:** Descriptive statistics of the attributes.

Attributes	Mean	Standard deviation
V1: Crosswalk usage is time-consuming	3.17	1.09
V2: Absence of guard rails on median	3.74	1.26
V3: Unsuitability of crosswalk location	3.57	1.15
V4: Feel unsafe to cross directly without crosswalk	3.95	1.39
V5: Inadequate marking and sign to locate crosswalk	3.74	1.00
V6: Poor entry access to crosswalk	3.94	1.05
V7: Difficulty crossing roads using crosswalk	3.97	1.09
V8: Inadequate lightning at night near crosswalk	4.11	1.01

Cronbach's alpha = 0.871.

## 4 Experimental Design, Materials and Methods

This study employed a quantitative approach to explore pedestrian crosswalk usage behavior. The survey was designed based on an extensive literature review [[5], [6], [7], [8], [9], [10], [11], [12], [13], [14], [15], [16], [17]], consultations with local transportation experts, and input from transport authority officials. A preliminary survey was conducted with 50 pedestrians from various occupations to refine the questionnaire. The main survey focused on eight attributes related to crosswalk behavior and demographic characteristics (Gender, Age, Crosswalk Usage Frequency, and Preference for Crosswalk Usage). Responses were collected using a 5-point Likert scale, (Strongly Disagree to Strongly Agree), a format commonly used in perception-based studies for its clarity [1].

The survey was carried out over three months at twelve important locations in Dhaka, Bangladesh. A total of 700 responses were collected by a team of five individuals under proper supervision. After data screening to remove unengaged respondents and outliers, the final sample size was 682, which is well above the minimum required sample size of 384 [1,2] . The footpaths near intersections and roundabouts in those survey areas were visited on various weekdays to ensure a diverse sample of pedestrians from across Dhaka City.

## Limitations

This dataset, used to study factors influencing pedestrian crosswalk usage behavior in Dhaka city, shows a gender bias, with females comprising only 9.24 % of the sample. This underrepresentation is due to socio-cultural dynamics in Bangladesh, where females are less likely to engage with unknown individuals and often refuse face-to-face surveys. Additionally, religious and cultural norms discourage females from interacting with survey conductors, especially if the conductor is an unknown male. This gender bias is a limitation of the study and is consistent with findings from other surveys in Bangladesh that also report low female participation (e.g., [18]). Despite this limitation, it is important to note that the factors influencing crosswalk usage—such as time consumption, safety, and accessibility—are not inherently gender-specific. Previous studies have shown that these factors impact pedestrian crosswalk usage behavior irrespective of gender [[19], [20], [21]]. Consequently, while the gender bias in our dataset is acknowledged, the insights derived remain valuable and relevant for understanding pedestrian crosswalk usage behavior in developing countries like Bangladesh.

Additionally, while our methodology provides valuable insights into factors influencing crosswalk usage, it is important to acknowledge a broader limitation inherent in self-reported data. Numerous studies have shown that there can be discrepancies between what users declare in questionnaires and their actual behavior [[22], [23], [24]]. This discrepancy suggests that user perceptions captured through questionnaires may not fully align with their real-world behavior at pedestrian crossings.

## Ethics Statement

Ethical review and approval were not required for the study on human participants in accordance with the local legislation and institutional requirements. All pedestrians participated in the survey willingly. All respondents consented to the survey and the use of the data for non-commercial research objectives. All data were collected anonymously.

## CRediT Author Statement

**Nazmus Sakib:** Conceptualization, Methodology, Software, Formal analysis, Writing-Original draft, Visualization, Data curation. **Tonmoy Paul:** Conceptualization, Writing- Reviewing and Editing. **Md. Tawkir Ahmed:** Conceptualization, Methodology, Supervision. **Khondhaker Al Momin:** Conceptualization, Investigation, Writing- Reviewing and Editing, Supervision. **Saurav Barua:** Conceptualization, Investigation.

## Data Availability

Dataset on Factors Influencing Pedestrian Crosswalk Usage Behavior in High-Density Urban Areas of a Developing Country (Original data) (Mendeley Data).
